# Universal Background Checks, Permit Requirements, and Firearm Homicide Rates

**DOI:** 10.1001/jamanetworkopen.2024.25025

**Published:** 2024-08-01

**Authors:** Michael Siegel

**Affiliations:** 1 Department of Public Health and Community Medicine, Tufts University School of Medicine, Boston, Massachusetts

## Abstract

**Importance:**

A loophole in US gun policy is that people can purchase guns from private sellers without going through any background check. Some states have addressed this loophole by requiring universal background checks for all gun sales, either at the point of sale or through a permit system; however, most studies on the effectiveness of universal background checks have not analyzed these 2 policy mechanisms separately.

**Objective:**

To assess the association of point-of-sale background check law and gun permit law, separately, with firearm homicide rates from 1976 through 2022 using the same methods and model specification.

**Design, Setting, and Participants:**

This cross-sectional study used a difference-in-differences, fixed-effects regression model to evaluate firearm laws and firearm homicide rates in 48 states from 1976 through 2022. Data were obtained for 48 states except New Hampshire and Vermont and were analyzed in January 2024.

**Exposures:**

Implementation of either the law requiring a universal background check at point of sale for all firearms without a permit or the laws combining universal background checks and a state permit requirement for all gun purchasers.

**Main Outcomes and Measures:**

Annual, state-specific rates of firearm homicide per 100 000 people.

**Results:**

From 1976 through 2022, 12 states adopted the universal background check laws without permitting requirements and 7 states implemented gun permit laws covering all firearms. The mean (SD) firearm homicide rate was 4.3 (0.1) per 100 000 people. Universal background checks for all firearms alone (without a state permitting system) were not associated with overall homicide rates (percentage change, 1.3%; 95% CI, −6.9% to 10.4%) or firearm homicide rates (percentage change, 3.7%; 95% CI, −5.3% to 13.6%). A law requiring a permit for the purchase of all firearms was associated with significantly lower overall homicide rates (percentage change, −15.4%; 95% CI, −28.5% to −0.01%) and firearm homicide rates (percentage change, −18.3%; 95% CI, −32.0% to −1.9%).

**Conclusions and Relevance:**

This cross-sectional study found that universal background checks alone were not associated with firearm homicide rates, but a permit requirement for the purchase and possession of firearms was associated with substantially reduced rates of firearm homicide. The findings suggest that combining universal background checks and permit-to-purchase requirements is an effective strategy for firearm-related fatality reduction.

## Key Points

**Question:**

Are US state laws requiring universal background checks for all gun purchases and/or laws requiring permits to purchase guns associated with reduced rates of firearm homicide?

**Findings:**

In this cross-sectional study of 48 states, permit requirements, but not universal background checks alone, were associated with reduced firearm homicide rates.

**Meaning:**

The finding suggests that to reduce firearm violence, a universal background check law must be paired with a permit requirement law for the purchase of all firearms.

## Teaser

This cross-sectional study evaluates the association of point-of-sale background check laws and state permit requirements with firearm homicide rates in 48 US states from 1976 through 2022.

## Introduction

A loophole in US gun policy is that people can purchase guns from private sellers without going through any background check. Therefore, state laws to require universal background checks for all gun purchases—even from private sellers—have been the mainstay of public health efforts to reduce gun violence in the US.^1^ There is evidence that state laws mandating universal background checks are associated with a substantial decrease in firearm homicide rates.^2,3,4,5,6,7,8,9^ However, there are 2 mechanisms by which background checks are implemented. Some states require background checks at the point of sale any time a firearm is purchased. Other states require anyone interested in purchasing or possessing a gun to first obtain a state permit, and a background check is conducted as part of the permitting process. Until recently, little distinction was made between these 2 mechanisms.

However, 2 studies examined the outcome of point-of-sale background check laws separately from the outcome of gun permit laws.^10,11^ The studies reported that point-of-sale background check laws were not associated with reduction in firearm homicide rates.^10,11^ In contrast, universal background checks conducted through gun permitting systems have been found to be consistently associated with significantly lower firearm homicide rates (eTable 1 in Supplement 1).^6,7,12,13,14,15,16,17,18,19^ A major limitation of the research on universal background checks is that few studies have simultaneously assessed the implications of point-of-sale background check only law and gun permit laws for firearm homicide rates using the same methods and model specifications. To date, only 2 such studies have been conducted.^12,20^

First, Crifasi et al,^20^ in a study of legislation implemented through 2015, found that laws requiring universal background checks at the point of sale but without a gun permit were associated with a 10% increase in firearm homicide rates in urban counties, whereas laws requiring permits to purchase a gun were associated with an 11% decrease in firearm homicide rates (eTable 1 in Supplement 1). Second, McCourt et al,^12^ in a study of legislation implemented through 2017, found that laws requiring point-of-sale universal background checks in Maryland and Pennsylvania were not associated with firearm homicide rates, whereas permit-to-purchase laws in Connecticut and Missouri were associated with significantly lower firearm homicide rates (eTable 1 in Supplement 1).

Because of newly available data, these relationships can now be modeled over a 47-year period from 1976 through 2022. Thus, the purpose of this study is to assess the association of point-of-sale background check law and gun permit law, separately, with firearm homicide rates from 1976 through 2022 using the same methods and model specification.

## Methods

### Study Design

This cross-sectional study used a panel data design with annual state-level data on firearm violence rates, state firearm laws, and time-varying state control variables for 48 of the 50 US states for 1976 through 2022. The Tufts University Institutional Review Board deemed this study exempt from review and informed consent requirement because it analyzed publicly available, deidentified datasets. The study followed the Strengthening the Reporting of Observational Studies in Epidemiology (STROBE) reporting guideline.

A difference-in-differences, fixed-effects linear regression was performed to leverage differences in the timing of universal background check and gun permit laws across states to examine whether implementation of these laws was associated with changes in outcomes that were substantially different from concurrent changes in states without these firearm laws. The outcomes examined were the age-adjusted total and firearm-related homicide rates for each state. The regressions included year and state fixed effects and controlled for a range of potential state-level confounding variables, including the presence of other firearm laws.

### Sample

**Figure 1.**
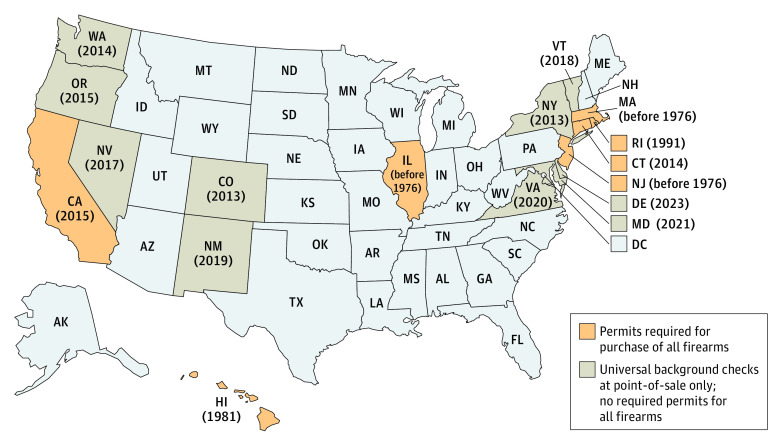
Universal Background Check and Gun Permit Laws in the US as of 2022 The year indicates when the law was enacted in the state.

The sample consisted of 48 states, 10 of which had universal background checks at the point of sale only (without a gun permit law) that covered all firearms as of 2022. An additional 7 states had laws requiring a permit to purchase any firearm. Figure 1 depicts the states with point-of-sale background check law only and states with permit requirement law as of 2022 along with the year in which the law was enacted.

### Data and Measures

The primary outcome variables were the annual firearm, nonfirearm, and total homicide rates in each state. Age-adjusted death rates were obtained from the Center for Disease Control and Prevention Web-based Injury Statistics Query and Reporting System (CDC WISQARS), which contains data from the death certificate–based National Vital Statistics System.^21,22^ Because the CDC suppresses death rates that are based on fewer than 10 decedents, 2 states for which data were missing for 10 or more years (New Hampshire and Vermont) were excluded. Data were extracted for 1980 through 2022. Data for 1976 through 1979 were obtained from a database made publicly available by Kang and Rasich,^23^ who scanned National Vital Statistics Reports and recorded age-adjusted firearm and nonfirearm homicide rates. A histogram revealed that the homicide rates were highly skewed. Therefore, for the present study, these rates were log transformed, which produced a distribution that approximated a normal distribution.

The independent variables were dummy variables representing the presence of a point of sale without a permit law and permit requirements for purchase of all firearms, coded as a 0 or a 1. Because it may take time for gun laws to affect population-level homicide rates, laws were modeled as being in effect during the second year after their enactment. For example, a law that went into effect in 2005 was modeled as being present in 2007. The status of firearm laws was obtained from the State Firearm Law Database,^24^ which was developed by searching statutes, legislative histories, and session laws for all 50 states using the Westlaw Edge research platform.^25^

A number of state-level factors were controlled for and were chosen a priori based on their association with homicide rates in prior studies.^5,6,7^ These control variables included the log of the total population, population density, nonhomicide violent crime rate, property crime rate, crude overall suicide rate, percentage of the population who identified as non-Hispanic Black individuals, poverty rate, unemployment rate, incarceration rate, and per capita alcohol consumption.

Race and ethnicity population data were used as a control variable because firearm homicide rates are known to be substantially higher in the Black population.^7^ If enactment of firearm laws is associated with the percentage of the Black population in a state, then this variable could confound the observed association between firearm laws and firearm homicide rates. Crime rates were obtained from the Uniform Crime Reports,^26^ demographic data from the American Community Survey 1-year estimates,^27^ alcohol consumption from the National Institute on Alcohol Abuse and Alcoholism,^28^ and incarceration rates from the Bureau of Justice Statistics National Prisoner Statistics series.^29^ Alcohol consumption data were not yet available for 2022 and were not reported for 1976; thus, data from the preceding year were used. Incarceration rates for 2022 were also not available, so the national change in incarceration from 2021 through 2022 was applied to each state. Poverty rates were not available from 1976 through 1979 and thus were linearly interpolated from the data for 1975 and 1980. Poverty data for 1975 and unemployment data for 1976 through 1979 were obtained from the 1980 Statistical Abstract of the United States.^30^ The percentage of Black residents in each state was also interpolated for 1976 through 1979 from 1975^31^ and 1980 data. Crude overall suicide rates were obtained from CDC WISQARS^21,22^ for 1980 through 2022 and from the Kang and Rasich database for 1976 through 1979.^23^

Because universal background check and gun permit laws may be more likely to be adopted by states that have enacted other state firearm laws that reduce firearm homicide, these other laws could confound any observed association between firearm laws and death rates. The most closely associated law enacted along with universal background check law is the may-issue concealed carry law, which gives law enforcement authorities discretion in approving applications for concealed carry permits. Thus, the presence or absence of a may-issue law was controlled for. In addition, 2 other state firearm laws were controlled for that have been shown in other studies to potentially affect homicide rates: (1) laws that prohibit firearm possession by people with a domestic violence restraining order and (2) laws that require prohibited persons to relinquish all guns in their possession.

Each observation in the dataset represented variables in a given state during a given year. Thus, these variables were time-varying factors within each state. Because there were 48 states and 47 years, the total number of observations in the dataset was 48 times 47, or 2256.

### Statistical Analysis

A difference-in-differences, fixed-effects linear regression model was used. Data analysis was performed in January 2024.

The formal model was as follows: ln(fh*_st_*) = β_1_(B*_st_*) + β_2_ (P*_st_*) + β_3_(X*_st_*) + y*_t_* + z*_s_* + e*_st_*, where ln(fh*_st_*) was the natural log of the homicide rate in state *s* at time *t*, B*_st_* was a dummy variable for the presence or absence of a point-of-sale universal background check law that covered all firearms without a gun permit law that covered all firearms, P*_st_* was a dummy variable for the presence or absence of a law that required a permit to purchase any firearm, X*_st_* was a vector of control variables, y*_t_* were year fixed effects, z*_s_* were state fixed effects, and e*_st_* was the error term. The use of a difference-in-differences model helped to ensure that changes in homicide rates associated with law implementation within a given state over time were being compared rather than absolute differences between homicide rates across states. This comparison helped address the possibility that states with lower homicide rates to begin with may have been more likely to enact stronger firearm laws.

The model relied on the assumption that homicide rates in a given year and state are independent of homicide rates in preceding years. This assumption may be violated because it is possible that homicide rates in a given year are affected by rates in the prior year, which is referred to as first-order serial autocorrelation.^32^ The model also assumed that variances of the error terms are homoskedastic (ie, equal across states and years). However, this assumption may be violated because there are differences in the population size across states, which is likely to introduce heteroskedasticity.^32^ The presence of serial autocorrelation or heteroskedasticity will yield inefficient estimates of regression coefficients and biased estimation of their SEs.^32^ To address this problem, a generalized least squares estimator designed for a linear regression in which the errors are serially correlated (ie, Prais-Winsten regression), accounting for both serial autocorrelation and heteroskedasticity in the data, was used.^32,33^ The Prais-Winsten regression transformed the error terms into serially uncorrelated errors,^32^ which was implemented in Stata (StataCorp LLC) using the prais command.^33^ In addition, robust SEs, which are unbiased even in the presence of serial autocorrelation or heteroskedasticity, were used.^33^ The full Stata syntax is shown in the eMethods and eTable 2 in Supplement 1. This approach has been used in several published econometric analyses of panel data with serial autocorrelation.^34,35,36,37,38^

The presence of serial autocorrelation in the initial and transformed models was checked using the Durbin-Watson statistic; a value close to 2 provides evidence of no serial autocorrelation.^33^ The initial model had a Durbin-Watson statistic of 0.94, demonstrating the presence of serial autocorrelation. After the Prais-Winsten transformation, the final model showed a Durbin-Watson statistic of 2.19, which was close enough to 2 to conclude that the errors were not serially correlated.^33^

Because the outcome variable was log transformed, the exponentiated regression coefficient minus 1 can be interpreted as the percentage change in the outcome associated with the implementation of the law. To ease the interpretation of regression coefficients for the control variables, each of them was standardized so that the exponentiated coefficient would yield the percentage change in the homicide rate associated with each 1-SD increase in the control variable.

As a falsification test, the association between the implementation of each type of law and the nonfirearm homicide rate was examined. If these laws were found to be associated with both lower firearm and nonfirearm homicide rates, it would cast doubt on the validity of the observed association.

## Results

### Descriptive Results

**Figure 2.**
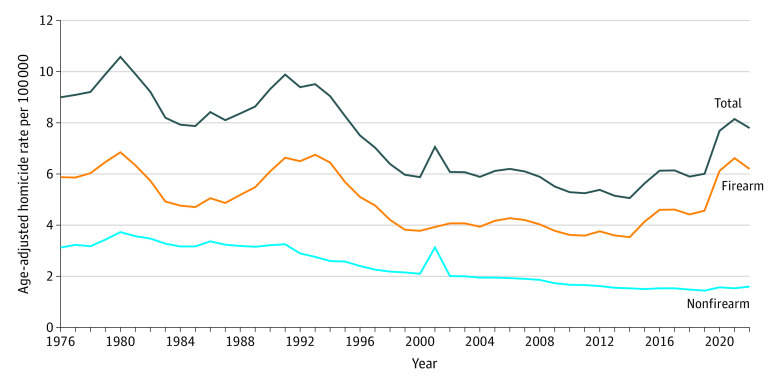
National Age-Adjusted Homicide Rates in the US From 1986 to 2022

In 2022, the age-adjusted firearm homicide rate ranged from a low of 1.0 per 100 000 people in Maine to a high of 18.5 per 100 000 people in Mississippi (Table 1). During the study period, 12 states adopted universal background check laws without permitting requirements, and 7 states implemented gun permit laws covering all firearms. The mean (SD) firearm homicide rate was 4.3 (0.1) per 100 000 people and ranged from 0.9 (0.4) per 100 000 people in New Hampshire to 11.3 (2.4) per 100 000 people in Louisiana. Nationally, the firearm homicide rate peaked in 1980, decreased in the mid-1980s before increasing again in 1991, and then rapidly declined until 2000 (Figure 2). After a small reduction from 2000 to 2014, there was a sharp increase through 2021, with a slight decrease in 2022. eTable 2 in Supplement 1 shows firearm homicide rate patterns just prior to the implementation of a state gun permit law. Firearm homicide rates were substantially lower in states implementing the legislation compared with all other states. However, the rates of change in firearm homicide rates just prior to enactment of a gun permit law were similar among half of the implementing states and moderately different in the other half (eTable 2 in Supplement 1).

**Table 1.** Firearm Homicide Rates and State Firearm Laws in 2022

State	2022 Status			
	Age-adjusted firearm homicide rate per 100 000	Universal point-of-sale background check for all firearms only, without required permit	Required permit for all firearms	Total No. of firearm laws
Mississippi	18.5	No	No	1
Louisiana	17.1	No	No	15
Alabama	13.4	No	No	7
New Mexico	11.2	Yes	No	17
Missouri	10.9	No	No	1
South Carolina	10.2	No	No	9
Arkansas	10.0	No	No	4
Georgia	9.8	No	No	1
Maryland	9.4	Yes	No	45
Tennessee	9.3	No	No	13
Illinois	9.1	No	Yes	50
North Carolina	7.7	No	No	17
Indiana	7.2	No	No	8
Arizona	7.2	No	No	7
Pennsylvania	7.0	No	No	25
Michigan	6.9	No	No	9
Ohio	6.8	No	No	10
Kentucky	6.8	No	No	1
Virginia	6.7	Yes	No	21
Oklahoma	6.5	No	No	4
Texas	6.2	No	No	9
Florida	6.0	No	No	19
Nevada	5.9	Yes	No	16
Alaska	5.4	No	No	2
Delaware	5.3	Yes	No	35
Colorado	5.3	Yes	No	24
Wisconsin	5.1	No	No	15
West Virginia	5.0	No	No	16
Kansas	4.6	No	No	6
California	4.4	No	Yes	79
Washington	4.1	Yes	No	36
Oregon	3.8	Yes	No	25
Montana	3.8	No	No	2
Connecticut	3.4	No	Yes	61
South Dakota	3.3	No	No	2
New York	2.9	Yes	No	57
New Jersey	2.8	No	Yes	58
North Dakota	2.8^a^	No	No	8
Minnesota	2.7	No	No	27
Nebraska	2.5	No	No	13
Rhode Island	2.5^a^	No	Yes	39
Idaho	2.2	No	No	1
Iowa	2.1	No	No	10
Wyoming	1.8^a^	No	No	5
Massachusetts	1.6	No	Yes	71
Hawaii	1.6^a^	No	Yes	55
Utah	1.5	No	No	11
Maine	1.0^a^	No	No	8

^a^ Data for 2021, as data for 2022 were suppressed.

### Regression Results

A universal background check for all firearms alone (without a state gun permitting system) was not associated with overall homicide rates (percentage change, 1.3%; 95% CI, −6.9% to 10.4%) or firearm homicide rates (percentage change, 3.7%; 95% CI, −5.3% to 13.6%) (Table 2). Laws requiring a permit for the purchase of all firearms were associated with significantly lower overall homicide rates (percentage change, −15.4%; 95% CI, −28.5% to −0.01%) and firearm homicide rates (percentage change, −18.3%; 95% CI, −32.0% to −1.9%). Gun permit laws were not associated with nonfirearm homicide rates.

**Table 2.** Percentage Change in Homicide Rates Associated With Firearm Laws

	Age-adjusted homicide rate, % change (95% CI)^a^		
	Overall	Firearm	Nonfirearm
**Control variables**			
Log of population	−5.6 (−21.3 to 13.2)	−9.8 (−26.8 to 11.1)	−12.0 (−24.1 to 2.1)
Population density	−46.9 (−54.9 to −37.5)	−10.8 (−25.1 to 6.3)	−45.1 (−51.5 to −37.9)
% Of Black individuals	258.0 (229.0 to 289.0)	277.8 (244.0 to 316.0)	62.6 (51.4 to 74.5)
Violent crime rate	5.1 (1.1 to 9.1)	8.2 (3.4 to 13.5)	9.9 (6.4 to 13.6)
Property crime rate	15.3 (10.7 to 20.2)	14.6 (9.0 to 20.5)	8.5 (4.8 to 12.3)
Incarceration rate	−8.4 (−11.7 to −5.0)	−7.0 (−11.0 to −2.9)	−9.1 (−11.9 to −6.3)
Poverty rate	0.4 (−1.8 to 2.6)	0.5 (−2.5 to 3.6)	−1.4 (−4.0 to 1.2)
Per capita alcohol	3.6 (−1.7 to 9.2)	1.4 (−4.6 to 7.8)	5.8 (0.9 to 11.1)
Unemployment rate	−0.2 (−2.7 to 2.2)	−0.5 (−3.6 to 2.8)	0.2 (−2.2 to 2.7)
Suicide rate	2.1 (−1.2 to 5.4)	3.3 (−1.3 to 8.1)	7.2 (3.5 to 11.2)
**Other gun laws**			
May-issue law	0.5 (−3.8 to 5.0)	−1.9 (−7.2 to 3.6)	3.4 (−1.3 to 8.2)
Restraining order law	0.7 (−3.7 to 5.3)	0.6 (−4.5 to 5.9)	−3.9 (−7.8 to 0.2)
Relinquishment law	−4.4 (−12.0 to 3.8)	−1.9 (−11.1 to 8.2)	−8.4 (−13.9 to −2.6)
**Universal background checks for all firearms only and permit requirement for purchase of all firearms**			
Universal background check for all firearms only (without required permit for purchase of all firearms)	1.3 (−6.9 to 10.4)	3.7 (−5.3 to 13.6)	2.9 (−2.6 to 8.8)
Required permit for purchase of all firearms	−15.4 (−28.5 to −0.01)	−18.3 (−32.0 to −1.9)	−4.5 (−14.4 to 6.6)

^a^ Percentage change in outcome variable associated with the firearm law or with each 1-SD increase in the independent variable.

Other factors that were associated with higher overall and firearm homicide rates were the percentage of Black individuals, violent crime rate, and property crime rate (Table 2). Higher incarceration rates were associated with lower overall and firearm homicide rates.

## Discussion

This study is among the few studies separately examining the association of firearm homicide rates with 2 methods by which states have implemented universal background checks for all firearm purchasers: point-of-sale background check and permit-to-purchase requirement. A difference-in-differences, fixed-effects regression covering 1976 through 2022 showed that laws requiring a point-of-sale background check alone were not associated with reductions in firearm violence. In contrast, laws requiring a state permit for anyone who wished to purchase a firearm were associated with an 18.3% reduction in firearm homicide rates and a 15.4% reduction in overall homicide rates.

These findings are consistent with a number of previous studies that did not find any significant reduction in firearm homicide associated with background check law alone^10,11,12,13,14,20,39^ and with numerous studies that found an association between state gun permit law and decreased rates of firearm homicide.^6,7,12,13,14,15,16,17,18,19^ The present study adds to the literature by (1) being only the third study to simultaneously investigate the association of both universal background check and gun permit laws with firearm homicide rates using the same methods and (2) analyzing 47 years of observational data (the longest time span of any study to date) on the outcome of state firearm laws.

The model specification appeared plausible since the regression coefficients for all control variables were in the expected direction (eg, higher violent crime and property crime rates were associated with higher homicide rates). In addition, in the falsification test, permit requirements were not associated with nonfirearm homicide rates but associated with only firearm homicide rates.

There are several possible reasons that a universal background check at the point of sale was not associated with reductions in firearm homicide, whereas a gun permit system had an association.^40^ First, unlike point-of-sale background checks, the requirement to obtain a permit to purchase a firearm generally requires interaction between the prospective buyer and law enforcement. Permit applications are typically submitted to a state law enforcement agency and require an appearance at the agency.^40^ Second, while background checks at the point of sale may require only a federal database check, gun permits require checks of state databases, which are more sensitive in picking up nonfelony crimes that are prohibitive for firearm ownership (eg, domestic violence misdemeanors, stalking offenses, misdemeanor violent crimes, and restraining orders).

### Strengths and Limitations

A strength of this study is that it used a difference-in-differences, fixed-effects model, which automatically controlled for all time-invariant differences between states and which compared changes within a state over time in response to the implementation of a law rather than differences between states at a given time. In this way, each state served as its own control so that a law enacted in a state with lower homicide rates to begin with was not necessarily deemed to be effective unless the change within that state after the law’s implementation was substantially different from changes in other states during the same period.

A limitation of this study is the possibility that states that enact a gun permit law are those with lower firearm homicide rates and/or those with firearm homicide rates that are already decreasing. Thus, an alternative potential explanation for the observed association between gun permit law and lower firearm homicide rates is reverse causation: rather than gun permit law playing a role in reduced firearm homicide, it may be that lower firearm homicide rates are a marker for states that are likely to adopt a gun permit law. Because of this limitation, this study cannot conclude that a causal association was found between a gun permit law and firearm homicide rates. These findings need to be confirmed in future studies, especially in research using alternative methods (eg, synthetic control). Nevertheless, a difference-in-differences approach helps defend against reverse causality. Since the difference-in-differences model compares each year’s homicide rate with the rate in the prior year, if the rates were falling before a law and continued to fall at the same pace after the law, the model would not capture any change.

Another limitation is that only firearm homicide rates, not suicide rates, were examined. There are several studies that found a relationship between state gun permit law and lower firearm suicide rates.^12,41^ This association should be explored in future studies.

## Conclusions

In this cross-sectional study of firearm laws, universal background checks alone were not associated with firearm homicide rates, but a permit requirement to purchase and possess a firearm was associated with substantially reduced rates of firearm homicide. This study provides new evidence that universal background checks alone may not be sufficient to prevent gun violence; however, combining this law with a permit-to-purchase requirement for all firearms could be an effective strategy for reduction of firearm-related fatalities.

## Notes

Supplement 1. **eMethods.** STATA Syntax Used to Run the Main Analysis **eTable 1.** Previous Studies Examining the Association of State Point-of-Sale Universal Background Checks or Permit Requirements With Firearm Homicide Rates **eTable 2.** Trends in Firearm Homicide Rates Prior to Implementation of State Gun Permitting Laws: Implementing States Compared to All Other States Without Permitting Laws Combined

Supplement 2.
Data Sharing Statement
