# Supplementary material for: Universal Background Checks, Permit Requirements, and Firearm Homicide Rates
Source: JAMA Netw Open. 2024 Aug 1;7(8):e2425025. doi: 10.1001/jamanetworkopen.2024.25025 (PMC11294962; doi:10.1001/jamanetworkopen.2024.25025)
Supplement: Supplement 1. — eMethods. STATA Syntax Used to Run the Main Analysis eTable 1. Previous Studies Examining the Association of State Point-of-Sale Universal Background Checks or Permit Requirements With Firearm Homicide Rates eTable 2. Trends in Firearm Homicide Rates Prior to Implementation of State Gun Permitting Laws: Implementing States Compared to All Other States Without Permitting Laws Combined [file jamanetwopen-e2425025-s001.pdf]

Supplemental Online Content

Siegel M. Universal background checks, permit requirements, and firearm homicide rates. *JAMA Netw Open*. 2024;7(8):e2425025. doi:10.1001/jamanetworkopen.2024.25025

eMethods. STATA Syntax Used to Run the Main Analysis

eTable 1. Previous Studies Examining the Association of State Point-of-Sale Universal Background Checks or Permit Requirements With Firearm Homicide Rates

eTable 2. Trends in Firearm Homicide Rates Prior to Implementation of State Gun Permitting Laws: Implementing States Compared to All Other States Without Permitting Laws Combined

This supplemental material has been provided by the authors to give readers additional information about their work.

© 2024 Siegel M. *JAMA Network Open*

eMethods. STATA Syntax Used to Run the Main Analysis

```
prais lnathr i.year i.states stdlnpopulation stddensity stdpctblack stdcrime stdproperty stdincarceration stdpoverty stdalcohol stdunemployment stdctsr L.lagmayissue L.lagdvro L.lagrelinquishment L.dummylagubconly L.dummylagpermit if fhstate==1, noconstant vce(robust)
```

prais invokes the Prais-Winsten regression method, a generalized least squares model to estimate unbiased regression coefficients and standard errors when error terms are serially correlated.

lnathr is the log of the age-adjusted total homicide rate

i.year inserts year fixed effects

i.states inserts state fixed effects

stdlnpopulation is the standardized log of the state population

stddensity is the standardized state population density

stdpctblack is the standardized percentage of Black residents in the state

stdcrime is the standardized non-homicide violent crime rate in the state

stdproperty is the standardized property crime rate in the state

stdincarceration is the standardized incarceration rate in the state

stdpoverty is the standardized poverty rate in the state

stdalcohol is the standardized per capita alcohol consumption in the state

stdunemployment is the standardized unemployment rate in the state

stdctsr is the standardized crude total suicide rate in the state

L.lagmayissue is the two-year lagged presence or absence of a "may issue" concealed carry law

L.dvro is the two-year lagged presence or absence of a law that prohibits gun possession by people subject to a domestic violence restraining order

L.relinquishment is the two-year lagged presence or absence of a law that requires all prohibited persons to relinquish their firearms as soon as they become prohibited from owning a firearm

L.dummylagubconly is the two-year lagged presence or absence of a universal background check law at point-of-purchase in the absence of a gun permitting requirement

L.dummylagpermit is the two-year lagged presence or absence of a law requiring a permit to purchase and possess any firearm

fhstate==1 restricts the analysis to 48 states (excluding New Hampshire and Vermont)

noconstant suppresses the intercept term

vce (robust) invokes robust standard errors

© 2024 Siegel M. *JAMA Network Open*

eTable 1. Previous Studies Examining the Association of State Point-of-Sale Universal Background Checks or Permit Requirements With Firearm Homicide Rates

Source (years covered)	Law examined	Summary of results
**Universal background checks at point of sale or through a permit (did not distinguish)**		
Kawano et al,[2] 2023 (2009-2018)	Universal background check at point of sale or through permit requirement	Association with lower firearm homicide rates
Sharkey and Kang,[3] 2023 (1991-2016)	Universal background check at point of sale or through permit requirement	Universal background checks were associated with significantly lower firearm homicide rates
Morrison et al,[4] 2021 (2000-2014)	Universal background check at point of sale or through permit requirement	Universal background checks were associated with a 0.6% decrease in firearm homicides in counties within the state
Siegel et al,[5] 2019 (1991-2016)	Universal background check at point of sale or through permit requirement	Universal background checks were associated with a 15% reduction in overall homicide rates
Siegel, Solomon, et al,[6] 2020 (1991-2016)	Universal background check at point of sale or through permit requirement	Universal background checks were associated with a 13% reduction in firearm homicide rates in large cities but were not associated with firearm homicide rates in suburban or rural areas
Knopov et al,[7] 2019 (1991-2016)	Universal background check at point of sale or through permit requirement	Universal background checks were associated with a 14% reduction in firearm homicide rates among both the White and Black populations
Kalesan et al,[8] 2016 (2010)	Universal background check at point of sale or through permit requirement	Universal background checks were associated with significantly lower rates of firearm homicide
Fleegler et al,[9] 2013 (2007-2010)	Universal background check at point of sale or through permit requirement	Universal background checks were associated with significantly decreased firearm homicide rates
**Universal background checks at point of sale only without a permit**		
Kagawa et al,[10] 2023 (1998-2019)	Universal background check at point of sale without permit requirement	No association with firearm homicide rates in Colorado, Delaware, Oregon, and Washington
McCourt et al,[11] 2020 (1985-2017)	Universal background check at point of sale without permit requirement	No association with firearm homicide rates in Maryland and Pennsylvania

© 2024 Siegel M. *JAMA Network Open*

Siegel, Goder-Reiser, et al,[12] 2020 (1976-2018)	Universal background check at point of sale without permit requirement	No association with the incidence or severity of mass public shootings
Webster et al,[13] 2020 (1984-2017)	Universal background check at point of sale without permit requirement	No association with the incidence of mass shootings
Castillo-Carniglia et al,[14] 2019 (1981-2000)	Universal background check at point of sale without permit requirement	No association with firearm homicide rate in California
Crifasi et al,[15] 2018 (1984-2015)	Universal background checks at point of sale without permit requirements	Point-of-sale background check only policies were associated with a 10% higher firearm homicide rates in urban counties
Kagawa et al,[16] 2018 (1981-2008)	Repeal of point-of-sale background check without permit requirement	No association with firearm homicide rates in Indiana and Tennessee
**Permit requirements**		
Crifasi et al,[17] 2023 (2015-2020)	Permit-to-purchase law	Gun permit laws were associated with a 28% lower rate of police shootings in Census tracts within the state
Liu et al,[18] 2022 (2000-2019)	State-level permit requirement	State permit requirements were associated with significantly lower rates of firearm homicide (1.79 per 100 000 population). Firearm homicide rates in the state were significantly higher (10.6 per 100 000 population) if neighboring states did not have a permit requirement.
McCourt et al,[11] 2020 (1985-2017)	State-level permit requirement in Connecticut and Missouri	Permit requirements were associated with a significantly lower firearm homicide rate (decreased by 28% in Connecticut after implementation of law; increased by 47% in Missouri after repeal of law)
Siegel, Goder-Reiser, et al,[12] 2020 (1976-2018)	State-level permit requirement	Permit requirements were associated with a 60% reduction in the odds of a mass public shooting occurring
Webster et al,[13] 2020 (1984-2017)	State-level permit requirements	Permit requirements were associated with a 56% reduction in the incidence of mass shootings
Siegel, Solomon, et al,[6] 2020 (1991-2016)	State-level permit requirement	Permit requirements were associated with a 20% reduction in firearm homicide rates in both urban and nonurban areas

Hasegawa et al,[19] 2019 (1994-2016)	State-level permit requirement	Repeal of permit requirements in Missouri was associated with a 22% increase in the firearm homicide rate
Knopov et al,[7] 2019 (1991-2016)	State-level permit requirement	Permit requirements were associated with an 18% reduction in firearm homicide rates among the White and Black populations
Crifasi et al,[15] 2018 (1984-2015)	State-level permit requirement	Permit requirements were associated with an 11% reduction in firearm homicide rates in urban counties
Rudolph et al,[20] 2015 (1984-2005)	Enactment of permit-to-purchase law in Connecticut (compared before and after enactment with that in 39 control states)	Implementation of law was associated with a 40% reduction in the firearm homicide rate
Webster et al,[21] 2014 (1999-2012)	Repeal of permit-to-purchase law in Missouri (compared before and after enactment with that in all other states)	Repeal of law was associated with a 23% increase in the firearm homicide rate

© 2024 Siegel M. *JAMA Network Open*

eTable 2. Trends in Firearm Homicide Rates Prior to Implementation of State Gun Permitting Laws: Implementing States Compared to All Other States Without Permitting Laws Combined

State	Year of implementation of permit law	Firearm homicide rate in year prior to implementation of permit law (per 100,000) – Implementing State	Firearm homicide rate in year prior to implementation of permit law (per 100,000) – All Other States Without Permit Laws Combined	Average change in firearm homicide rate during five years prior to law implementation (per 100,000) – Implementing State	Average change in firearm homicide rate during five years prior to law implementation (per 100,000) – All Other States Without Permit Laws Combined
Hawaii	1981	3.52	5.63	-0.58 per year	-0.58 per year
Rhode Island	1990	1.65	4.38	+0.11 per year	+0.08 per year
Connecticut	2014	1.90	3.60	-0.15 per year	-0.05 per year
California	2015	3.14	3.56	-0.13 per year	-0.00 per year

© 2024 Siegel M. *JAMA Network Open*
