# Supplementary material for: Universal Background Checks, Permit Requirements, and Firearm Homicide Rates
Source: JAMA Netw Open. 2024 Aug 1;7(8):e2425025. doi: 10.1001/jamanetworkopen.2024.25025 (PMC11294962; doi:10.1001/jamanetworkopen.2024.25025)
Supplement: Supplement 2. — Data Sharing Statement [file jamanetwopen-e2425025-s002.pdf]

Data Sharing Statement

Siegel. Universal Background Checks, Permit Requirements, and Firearm Homicide Rates. *JAMA Netw Open*. Published August 01, 2024. doi:10.1001/jamanetworkopen.2024.25025

Data
Data available: Yes
Data types: Data (not involving human participants)
How to access data: mike.siegel@tufts.edu
When available: With publication

Supporting Documents
Document types: None

Additional Information
Who can access the data: Anyone requesting the data
Types of analyses: For any purpose
Mechanisms of data availability: Email; no restrictions
Any additional restrictions: None
